# Moderate manipulation to somatosensory feedback does not affect Libet-style intentional action

**DOI:** 10.1007/s00426-025-02178-1

**Published:** 2025-09-08

**Authors:** Yu Hei Shum, Carl Michael Galang, Marcel Brass

**Affiliations:** Berlin School of Mind and Brain & Department of Psychology, Humboldt-Universität zu Berlin, Berlin, Germany

## Abstract

The existence of free will has been called into question by Benjamin Libet’s seminal experiment, who argued that our conscious decision is preceded by an unconscious decision reflected in the readiness potential (RP). Alternatively, it has been argue that the RP rather reflects a decision process in which different signals accumulate until they reach the intention threshold, at which point an agent experience their intention simultaneously. This raises the question what type of signal is accumulated given that no external information is provided. Recent studies suggest that various interoceptive signals, such as the respiratory and cardiac cycles, guide our arbitrary decisions. We hypothesized the somatosensory feedback from the afferent system could be another potential source, and tested this by inducing numbness in participants’ hands. If somatosensory feedback is one source of information in arbitrary decisions, participants should take longer to reach the decision threshold. Contrary to our hypotheses, Bayesian analyses revealed decisive evidence supporting the null hypothesis. These findings suggest that somatosensory feedback may not substantially contribute to arbitrary decisions, and alternative explanations have been proposed to account for the results.

**Supplementary Information:**

The online version contains supplementary material available at 10.1007/s00426-025-02178-1.

## Introduction

The cognitive and neural mechanism behind voluntary action and its implication to the existence of free will have intrigued researchers in multiple disciplines. The well-known Libet experiment (Libet et al., 1983) is one of the attempts to investigate these questions. In a classical set up, participants were presented with a clock featuring a dot rotating around the clock face. Participants were instructed to fixate on the clock and press a key whenever they wanted. After the keypress, they reported the moment they decided to press the key based on the position of the dot. A common intuition would assume that if we have “free will”, brain signals reflecting the decision should only occur after the decision to press the key was made. However, Libet et al. found the readiness potential (RP), a neural correlate of voluntary action, preceded the self-reported intention onset by hundreds of milliseconds.

**Fig. 1:**
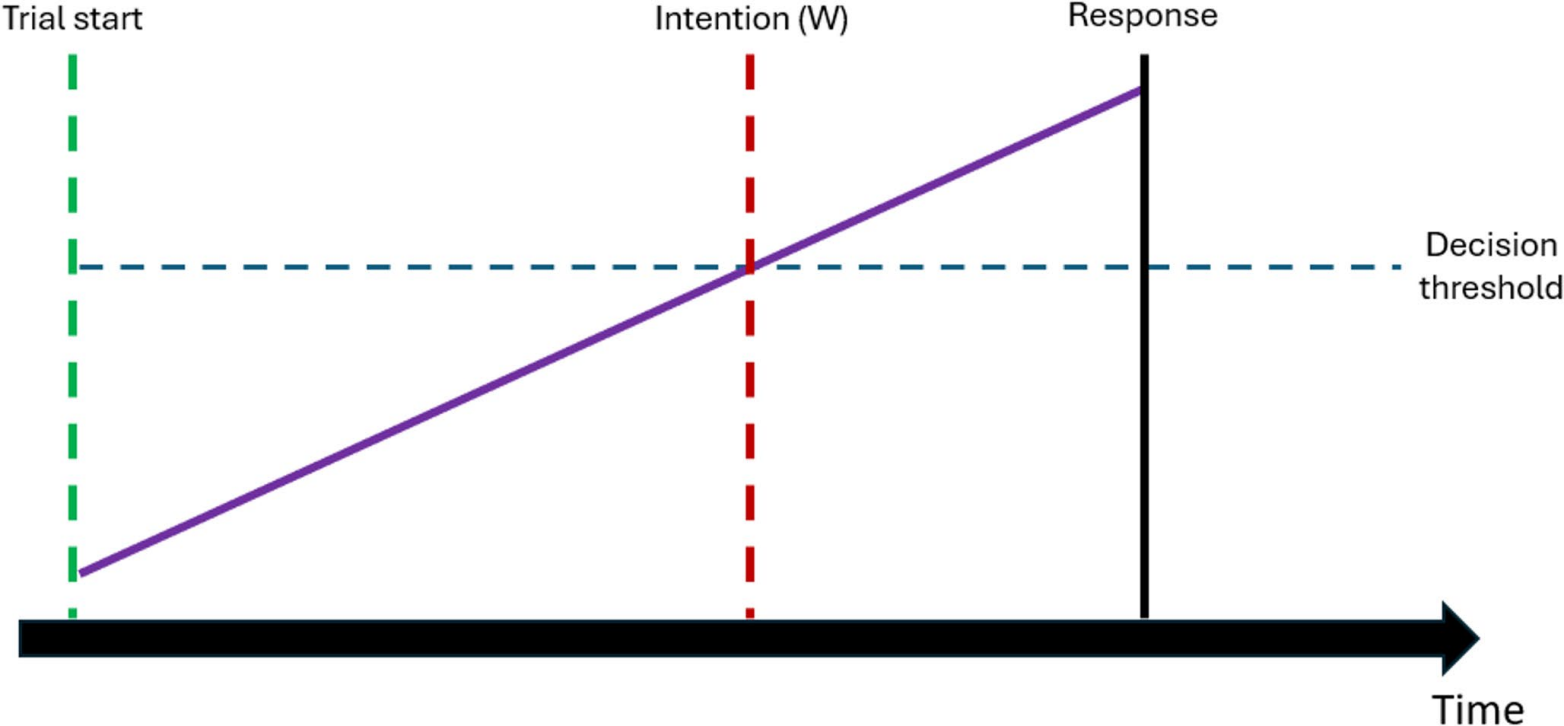
Illustration of the ITB. The decision signal (purple line) starts to accumulate at the beginning of a trial. When it exceeds the decision threshold (blue dashed line), participants experience their intention

**Fig. 2:**
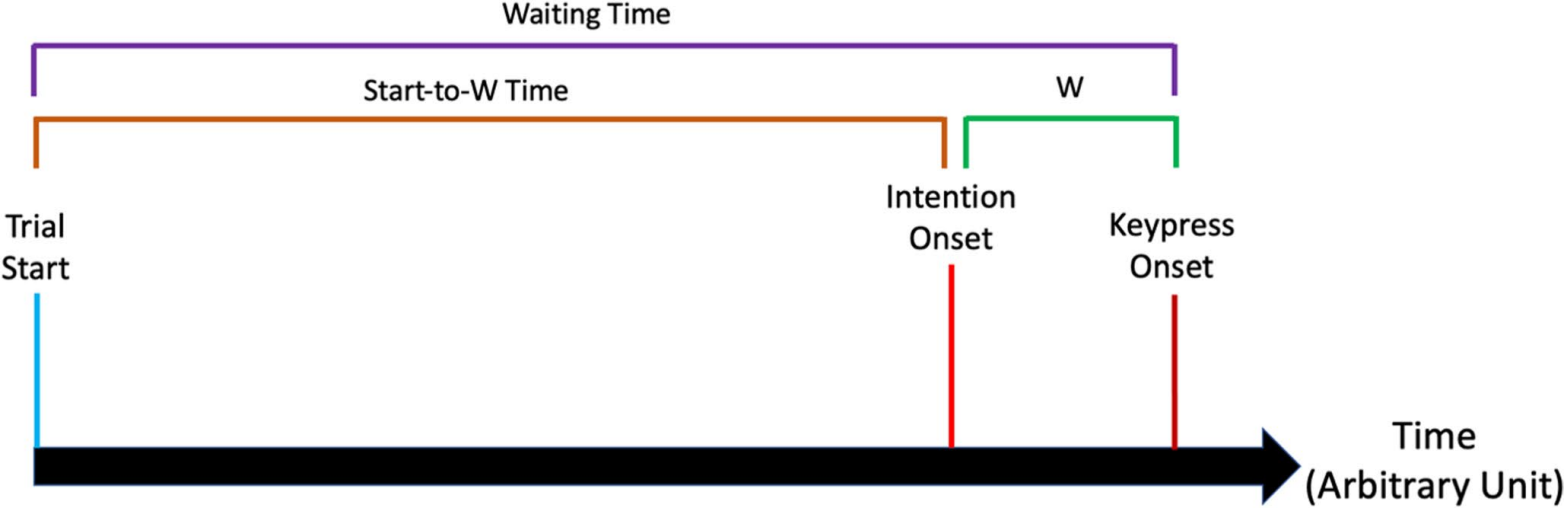
Summary of the measures in the Classical Libet task. Traditionally, the W is measured relative to the action onset. If the reported intention precedes the action onset, the W will be a negative value

While Libet and colleagues (1983) argued that the RP is a marker of the unconscious decision of an agent’s action occurring before s/he experienced a conscious intention, others have argued that the RP rather reflects the decision process itself (e.g. Bode et al., 2014, Brass et al., 2019; Schurger et al., 2012). This was captured formally in integration-to-bound models of intentional action (ITB; Bode et al., 2014; Brass et al., 2019; Schurger, 2018; Schurger et al., 2012, 2021; Fig. 1). According to the ITB, the RP reflects the process of accumulating evidence towards a decision threshold. The “Conditional Intention and Integration to Bound Model” (COINTOB; Brass et al., 2019), a variant of the ITB, proposes that participants first specify the parameters of the decision process based on the required task (e.g., the instruction of the experiment). These parameters include the thresholds of the decision bounds, as well as the signal accumulation rate (Shum et al., 2024). Then, participants start accumulating information. Once the accumulated information reaches the threshold of the intention bound, participants generate their proximal intention (measured as the Start-to-W Time, see Fig. 2). However, this is not yet the end of the decision-making process. The accumulation of information continues until the point-of-no-return, resulting in the action (measured as the Waiting Time, see Fig. 2). In this case, the time between experiencing the intention and performing the action is referred to as W in the literature (see Fig. 2). On the other hand, the decision signal may drop below the decision threshold, leading to a veto of the intended action.

In principle, the decision-making process in the Libet-style experiments and other evidence-based decision-making contexts, such as perceptual decision, can both be explained by integration to bound models. The only difference between the two decision types is simply the specific information being accumulated (Bode et al., 2014; Brass et al., 2019; Roskies, 2010). When making a perceptual decision, task-relevant information, such as the motion direction of stimuli in a random dot motion task, is accumulated, such that one can perform the task optimally. In an arbitrary decision- making situation including the Libet-style experiment, however, there is no task-relevant information to guide participant’s decision. This raises the question about the type of information that is accumulated here. It has been suggested that in the absence of exteroceptive information, interoceptive information are accumulated instead (Brass et al., 2019; Park et al., 2020; Schurger & Uithol, 2015; Schurger et al., 2012, 2016, 2021). Schurger and Uithol (2015) described this hypothesis as a paradigm shift in the cognitive neuroscience of decision-making. Traditionally, decision-making has been viewed as a unidirectional relationship from the brain to the body, where the brain decides and sends the decision to other body parts for execution. This hypothesis, on the other hand, proposes that the relationship between the brain and the body is dynamic and bi-directional, which expands the fundamental building blocks of decision-making via suggesting that body parts are not merely “executers” but also “deciders”.

Two types of information might be relevant for internally-guided decisions: information about the autonomic nervous system such as heart rate or breathing and afferent information from the motor system such as muscle tension as indicated by somatosensory feedback. Recent studies supported the idea that interoceptive signals from the autonomic nervous system guide our arbitrary decisions. Park and colleagues (2020) found that the timing of keypresses in the Libet task was coupled with the respiratory cycle, with participants more likely to execute the action during expiration. Interestingly, this coupling was not found when participants were responding to external stimuli (i.e., a perceptual decision), further supporting the idea that internal body information carries more weight in arbitrary decisions rather than in perceptual decisions. In a separate study, Park and colleagues (2022) showed that arbitrary timing of initiating a mental imagery was also coupled with the respiratory cycle, with participants’ self-reported onset of mental imagery more likely to occur during expiration. In both studies, neural measures indicated that the readiness potential (RP) was more negative during the expiration phase of respiration. Taken together, these findings suggest that respiratory signal are associated with arbitrary decisions in the Libet-style contexts, but not with perceptual decisions, which are primarily guided by sensory evidence.

Apart from respiratory signals, another potential source of influence on our arbitrary decision in Libet-style experiment is the signal from our motor system (Schurger & Uithol, 2015), with the somatosensory feedback in the afferent system as one of the candidates. Primary afferent neurons encode information about the physical states of the environment as well as the tissues they innervate (Furness et al., 2004; Martin, 1981). With this information, we predict the sensory consequences of our actions, compare them with the actual sensory outcomes, and then adjust our bodily states accordingly to fulfil our goals (Wolpert & Flanagan, 2001). Under this mechanism, the somatosensory signal from the effector should be more attended, since it is more relevant for generating prediction of sensory outcome. In line with this idea, Juravle and Deubel (2009) found that participants were more sensitive to tactile stimuli presented to the effector hand during motor preparation compared to the resting hand. At the neural level, a tactile probe delivered to the effector hand elicited stronger N140, an event-related potential associated with early somatosensory processing, than that delivered to the resting hand (Eimer et al., 2005). Taken together, these findings support the idea that the somatosensory feedback from the effector hand is critical for forward modelling during the preparation of intentional actions. Assuming that the somatosensory signal is a source of information being accumulated during intentional action, disruption to the somatosensory feedback from the effector hand should result in increased Start-to-W Time and Waiting Time.

One way to study this hypothesis is to induce a sense of numbness in participants’ hands by manipulating the temperature of the hand (Kammers et al., 2011; Mackworth, 1953, 1955; Mills, 1956). To achieve this, the cold pressor task, in which participants immerse their hands and forearms into cold water, is an effective and ethical method (Birnie et al., 2011). The cold pressor task has been found to elicit a strong somatosensory sensation, as well as triggering a strong activation in somatosensory cortex contralateral to the effector (Ray et al., 2019; Richardson et al., 2013), which is also a crucial region for generating the efferent copy for the forward modelling of action (Christensen et al., 2007; Gale et al., 2021).

In the decision-making domain, previous studies have shown that participants evidence-based decisions can be altered after experiencing the cold pressor task, including changes in visual and auditory stimulus detection tasks (Dizmen et al., 2015), risk-taking decisions in the Balloon Analogue Risk Task (Lighthall et al., 2012), regret in counterfactual context (Wu et al., 2021), and decisions to pursue long-term reward (Byrne et al., 2019). Since in the COINTOB model we assume that the somatosensory feedback plays a stronger role in arbitrary decisions than externally-guided action, one should expect that the cold-pressor task also affect arbitrary decisions in Libet-style experiment.

The current study investigated whether manipulating somatosensory feedback in the afferent system would affect participants’ arbitrary decisions in the Libet-style experiment. Participants’ somatosensory feedback in their right hands was manipulated by the cold pressor task (i.e., the Cold Right Condition). They then performed the Classical Libet task with the same hands immediately after immersion. To measure their baseline performance, participants immersed their right hands and forearms in warm water and performed the same task (i.e., the Warm Right Condition). We hypothesized that after immersion in cold water, participants’ generation of intention and intentional action would be affected by numbness in their somatosensory feedback, resulting in longer Start-to-W Time and Waiting Time due to a prolongation of the evidence accumulation process.

The cold pressor task has been found to elicit changes across various physiological and psychological domains beyond somatosensory feedback, such as stress (Lovallo, 1975; Roberts et al., 2015), pain (von Baeyer et al., 2005; Birnie et al., 2012), and changes in blood pressure, breathing rate, and heart rate (Richardson et al., 2013). Furthermore, it has been found to modulate neural activity in a wide range of brain regions, including the contralateral somatosensory cortex, anterior insular cortex, posterior cingulate cortex, hippocampus, cerebellum, and hypothalamus (Richardson et al., 2013). These physiological and psychological changes may also influence intentional action (e.g., Park et al., 2020; Germanova et al., 2025). Since our primary interest was the effect of the cold pressor task on somatosensory feedback from the effector hand, we included a control condition in which participants immersed their left hands and forearms in cold water before performing the Classical Libet task with their right hands (i.e., the Cold Left Condition). Given the role of forward modelling in action, we hypothesized that participants’ Start-to-W Time and Waiting Time would be longer in the Cold Right Condition than those in the Cold Left Condition.

## Methods

### Participants

The current experiment was pre-registered on *AsPredicted* (https://aspredicted.org/NNR_2P5). All data, analysis scripts, and experiment programs are available on the Open Science Framework (OSF; https://osf.io/vduar/?view_only=fd0614f10e1e4c3b9d6d111de6ac6021). The pre-registered sample size was 78, based on power analysis (G*Power 3.1, Faul et al., 2007) assuming a medium effect size (analysis specification: parameters estimated η_p_² = 0.06, with expected power 0.8, number of groups: 1, number of measurements : 3, nonsphericity correction : 1, effect size specification was set “as in SPSS” with η_p_² = 0.06 converted to f = 0.25). Participants were excluded if they met at least one of the following pre-registered exclusion criteria: (1) Participants who admitted that they always or often pre-planned their movement, (2) Participants with the standard deviation (SD) of keypress time lower than 100ms, (3) Participants with a variance of clock hand position when they press the key lower than 20 in at least one of the conditions, and (4) Outliers of the W and Waiting Time across participants based on boxplot. Values lower than the lower hinge minus 1.5*Interquartile range (IQR) or higher than the upper hinge plus 1.5*IQR were considered as outliers. The second and third criterion was applied as the low variance in Waiting time could be a sign of pre-planning their decision (e.g., always pressing the key whenever certain number of clock hand rotations were passed, or whenever the clock hand hits certain part of the clock). We collected the initial sample of 78 participants, 7 participants were rejected based on the aforementioned pre-registered exclusion criteria. Following the pre-registered protocol 7 more participants were collected as replacements. Therefore, the final sample size was the pre-registered *n* = 78 (20 males, 58 females). The mean age was 25.3 (SD: 4.37; range: 19–35).

Ethics approval was granted by the Ethics Committee of Humboldt Universität zu Berlin (reference number: 2023-01) before the experiment. All participants provided informed consent before the experiment. They were informed that their participation was voluntary, and all their responses would be processed and stored anonymously. Participants received compensation of 10€ for their participation.

### Design

**Fig. 3:**
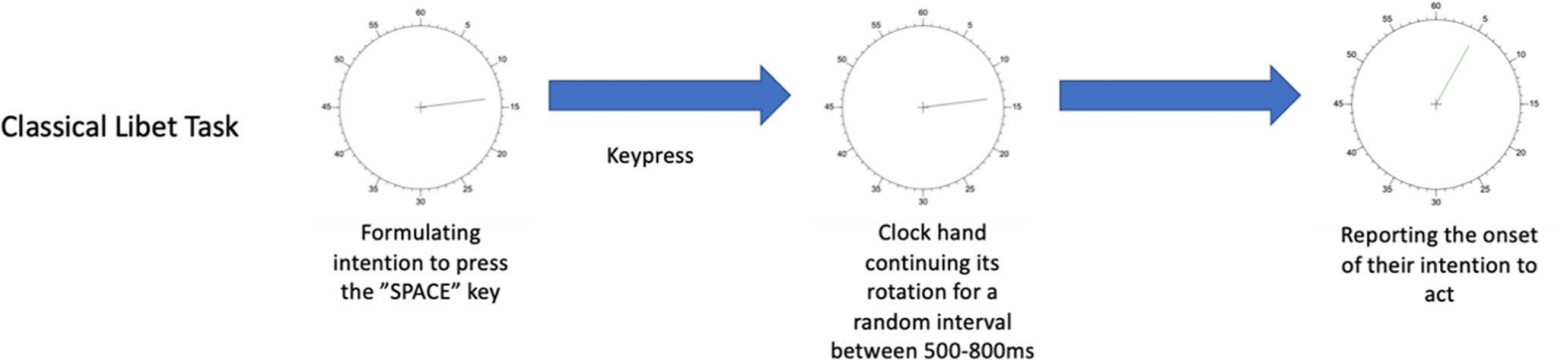
Paradigm of the Classical Libet task. Participants pressed the “SPACE” key whenever they decided and reported the onset of their intention

The experiment consisted of two tasks, the Classical Libet task (Fig. 3; Libet et al., 1983) and the cold pressor task. All the tasks were programmed with the Psychopy toolbox (version 2022.1.3; Peirce, 2007) in Python.

Each trial of the Classical Libet task clock face began with the appearance of a fixation cross (visual angle: 3°), followed by the display of a Libet clock (visual angle: 117°) with a 500ms delay. The clock hand started its clockwise rotation as soon as it appeared. The initial position of the clock hand was random, and it took 2560ms to complete one full rotation around the clock face. Participants fixated on the cross in the middle of the clock and waited for the clock hand to complete a full rotation. Then, they had to spontaneously decide when to press the “SPACE” key with their right index finger, and immediately execute the movement after making the decision. After the keypress, the clock hand continued its rotation for a random interval between 500 and 800ms (with 20ms as a step) before disappearing. A controllable clock hand then immediately reappeared. Participants reported the moment they experienced their own intention to press the key via indicating the position of the clock hand at that moment. The trial ended once the report was made. Participants were instructed to press the key before the third rotation of the clock hand ended and not to preplan their decisions. There were a total of 9 blocks of the Classical Libet task, with 20 trials in each block.

In the cold-pressor task, participants submerged their hand and forearm into a bucket of cold water (10 °C, +/- 1 °C) for 20 s. The water temperature was relatively high compared to other studies using the cold-pressor task (e.g., Fanninger et al., 2023). Different from most experiments in which participants only performed the cold-pressor task once, participants in the current study had to perform the cold-pressor task six times in total (three times for each hand, see below for details). We did not want their experience of pain to be too strong, as it might mask the potential effect of the somatosensory feedback on intentional action. After a few pilot sessions, we decided to use 10 °C as the water temperature based on participants’ self-reported degree of pain and numbness in the hand. Regarding the duration of the cold-pressor task, we initially decided to administer the task for 30 s instead of 20. However, during the pilot study, most participants reported experiencing strong pain, particularly in the later trials of the task. Therefore, we reduced the duration to 20 s for our cold-pressor manipulation.

There were three conditions in the cold-pressor task. In the Cold Right Condition, participants performed the cold-pressor task with their right hand. In the Cold Left Condition, participants performed the cold-pressor task with their left hand. In the Warm Right Condition, participants also performed the task with their right hand, but the water temperature was at a comfortable temperature (30 °C, +/- 1 °C). Each condition was repeated three times in a block procedure. The order of the blocks for each condition was fully counterbalanced across participants.

### Procedures

After giving their informed consent, participants were given written and verbal instruction for the tasks and completed two practice blocks. The procedures for the two practice blocks were the same as those in the Classical Libet task, with one exception: in the first practice block, participants were instructed to report the moment when they pressed the key (i.e., the M judgment) instead of the W judgment. The practice block with the M judgment was included because previous studies (Dominik et al., 2017; Sanford et al., 2020) have shown that participants could better separate the W judgment from the M judgment after this practice. There were 5 trials in each practice block. Participants received feedback only when they pressed the key too early (i.e., earlier than the end of the first clock hand rotation), or if they did not press the key before the end of the third clock hand rotation. No feedback was provided during the actual experiment.

**Fig. 4:**
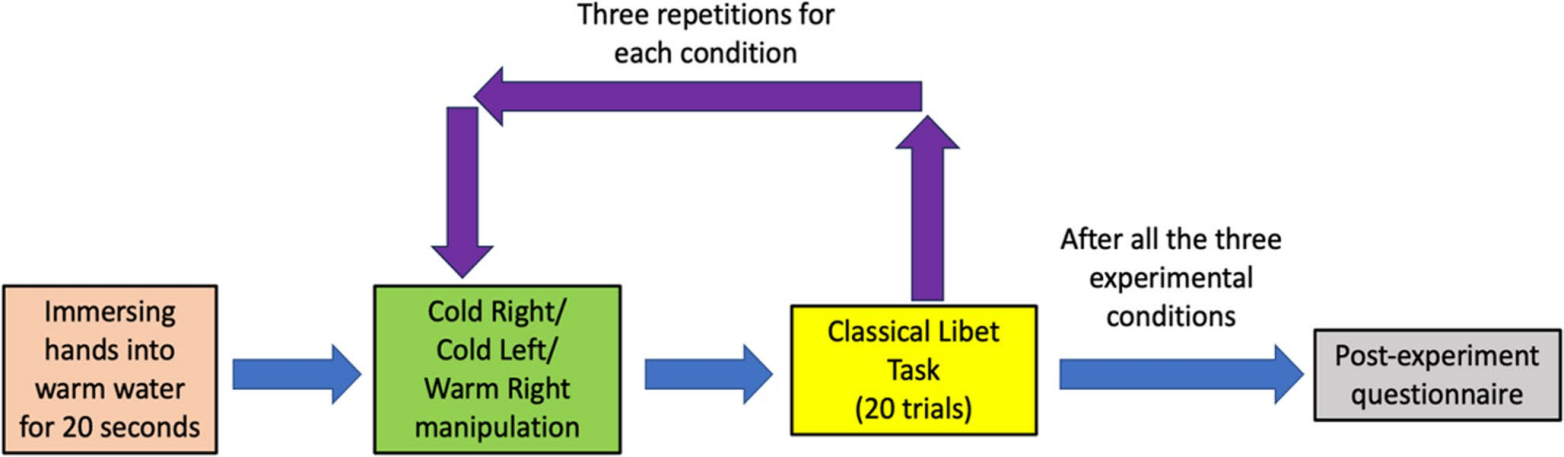
Timeline of the experiment procedure

The experimental procedures after the practice were summarized in Fig. 4. Immediately following the practice, the participants began the first experimental block by immersing their hands and forearms into warm water (30 °C, +/- 1 °C) for 20 s. The immersion into the warm water was only included before the first experimental block of each experimental conditions. They then dried their hands and performed the cold-pressor procedure for one of the experimental conditions. After 3 repetitions of the same experimental condition blocks, participants again immersed their hands and forearms in warm water before starting another experimental block. Once the last experimental block was finished, participants reported the frequency (Never, Seldom, Sometimes, Often, Always) in which they (1) pre-planned when to press the key, and (2) made their decision based on particular positions of the clock hand (i.e., coupling their intention to press the key with a specific clock hand position, such as pointing at “30”). Additionally, participants reported their subjective intensity of pain during the cold-pressor task using a 0 to 10 NRS scale (Hawker et al., 2011; Williamson & Hoggart, 2005) at the end of the experiment. Zero indicated “No Pain” while ten indicated “Worst Possible Pain”.

### Pre-registered analysis plan

We also calculated the W and the Start-to-W Time (Fig. 2) based on the behavioral data. First, the W for each trial was determined by comparing the clock hand position at the time of the keypress with the self-reported clock hand position based on participant’s experience of intention. We calculated the W as the closest distance between the keypress time and the reported intention onset. For instance, if the participant pressed the key at 1 o’clock and reported the intention onset at 11 o’clock, the W would be −426.67 ms (since it took 2560/12 = 213.33ms for the clock to travel from one “hour” to the next), rather than 2133.33 ms. Similarly, if the participant pressed the key at 1 o’clock and reported the intention onset at 3 o’clock, the W would be + 426.67 ms. Trials with outliers (defined as values lower than the lower hinge minus 1.5*IQR or higher than the upper hinge plus 1.5*IQR) were calculated individually and removed before the analysis.

We further calculated Start-to-W Time by adding W to Waiting Time (Shum et al., 2024). Since the W measured how much earlier the intention preceded the keypress (thus it was usually a negative value), addition was applied instead of subtraction. This method was preferable to subtracting the absolute value of the W from the Waiting Time, as it theoretically allowed the Start-to-W Time to exceed the Waiting Time when participants reported their intention to be later than their keypress.

After calculated the two measures, we conducted Bayesian repeated measures ANOVAs with Condition (Cold Right, Cold Left, Warm Right) as the factor for each of the three behavioral measures (Start-to-W Time, W, and Waiting Time), along with follow-up tests to compare each behavioral measure in pair-wise contrasts. In the pre-registration we mentioned that we would conduct the analyses in both the Frequentist and Bayesian approaches. This was aim to examine whether the results were consistent between two analytic approaches. Our results did not change based on the analytic approach. Therefore, only the Bayesian results were reported to maintain the conciseness of the paper. The default priors in Jamovi software (2022, version 2.3; Şahin & Aybek, 2019), was used to calculate the BF10. We interpreted the BF10 based on the guideline provided by Jeffreys (1998), Lee and Wagenmakers (2014) (see also; Schönbrodt & Wagenmakers, 2018). Specifically, a BF10 larger than 3 was considered to provide evidence supporting the alternative hypothesis, whereas a BF10 smaller than 0.3 was considered to provide evidence supporting the null hypothesis.

Although we have fully counterbalanced the orders of the conditions, we still wanted to examine whether the potential effect of the cold-pressor task manipulation on intentional action varied based on the order of the conditions. To address that, a pre-registered Bayesian repeated measures ANOVA with Condition (Cold Right, Cold Left, Warm Right) and Order (the 6 counterbalance sequences) as factors was conducted for each of the behavioral measure. Again, a BF10 larger than 3 for the interaction term would be considered as evidence suggesting that the strength of the cold-pressor manipulation to the parameters of the intentional action indeed varied with the orders of the conditions. Conversely, a BF10 smaller than 0.3 would be considered as evidence supporting a consistent effect strength across the counterbalancing orders.

### Exploratory analysis plan

#### Effect across time

We first explored whether the potential effect of the cold-pressor task manipulation on our intentional action would decay over time. To address this, we split each experimental block in half and performed the Bayesian repeated measures ANOVAs with Time (First Half of Each Block, Second Half of Each Block) and Condition (Cold Right, Cold Left, Warm Right) as factors for each of the three behavioral measures (Start-to-W Time, W, and Waiting Time). If there was evidence supporting the alternative hypothesis (i.e., a BF10 larger than 3) for the two-way interaction term (Condition*Time), follow-up tests were conducted to compare each behavioral measure in pairwise contrasts for both the first half and the second half separately. In total, there would be 6 follow-up tests (i.e., Cold Right First half vs. Cold Left First half, Cold Right First half vs. Warm Right First half, Cold Left First half vs. Warm Right First half, Cold Right Second half vs. Cold Left Second half, Cold Right Second half vs. Warm Right Second half, Cold Left Second half vs. Warm Right Second half).

#### Individual sensitivity to the Manipulation

Participants may have different sensitivities to the cold-pressor manipulation. To address this, we conducted a Bayesian repeated measures ANOVA with the factors Condition (Cold Right, Cold Left, Warm Right) and Pain Rating (the pain ratings measured at the end of the experiment; see Procedures for details). Participants were grouped into High Pain and Low Pain categories based on the median pain rating. The underlying assumption of this analysis was that if some participants were more sensitive to the cold-pressor manipulation, they should also experience stronger pain. Consequently, the effect of the cold-pressor task on our dependent measures should depend on subjective pain intensity.

#### Single-trial level model

Previous studies have shown that behavioural data in Libet-style experiment can be modelled with signal accumulation models (Schurger et al., 2012; Schurger, 2018; Trovò, 2021; Fig. 1). We extended this idea by fitting the Waiting Time with a one-bound signal accumulation model using the Shifted Wald distribution (Anders et al., 2016) and comparing the model parameters across experimental conditions. We were specifically interested in two model parameters: the rate of signal accumulation (α) and the decision threshold (γ). After modelling these parameters for each condition with each participant, we performed the Bayesian repeated measures ANOVAs with Condition (Cold Right, Cold Left, Warm Right) and Time (First Half, Second Half) as factors. Again, follow-up tests were only done if there was evidence supporting the alternative hypothesis (i.e., a BF10 larger than 3) for the two-way interaction term (Time*Condition).

## Results

### Pre-registered analysis

**Fig. 5:**
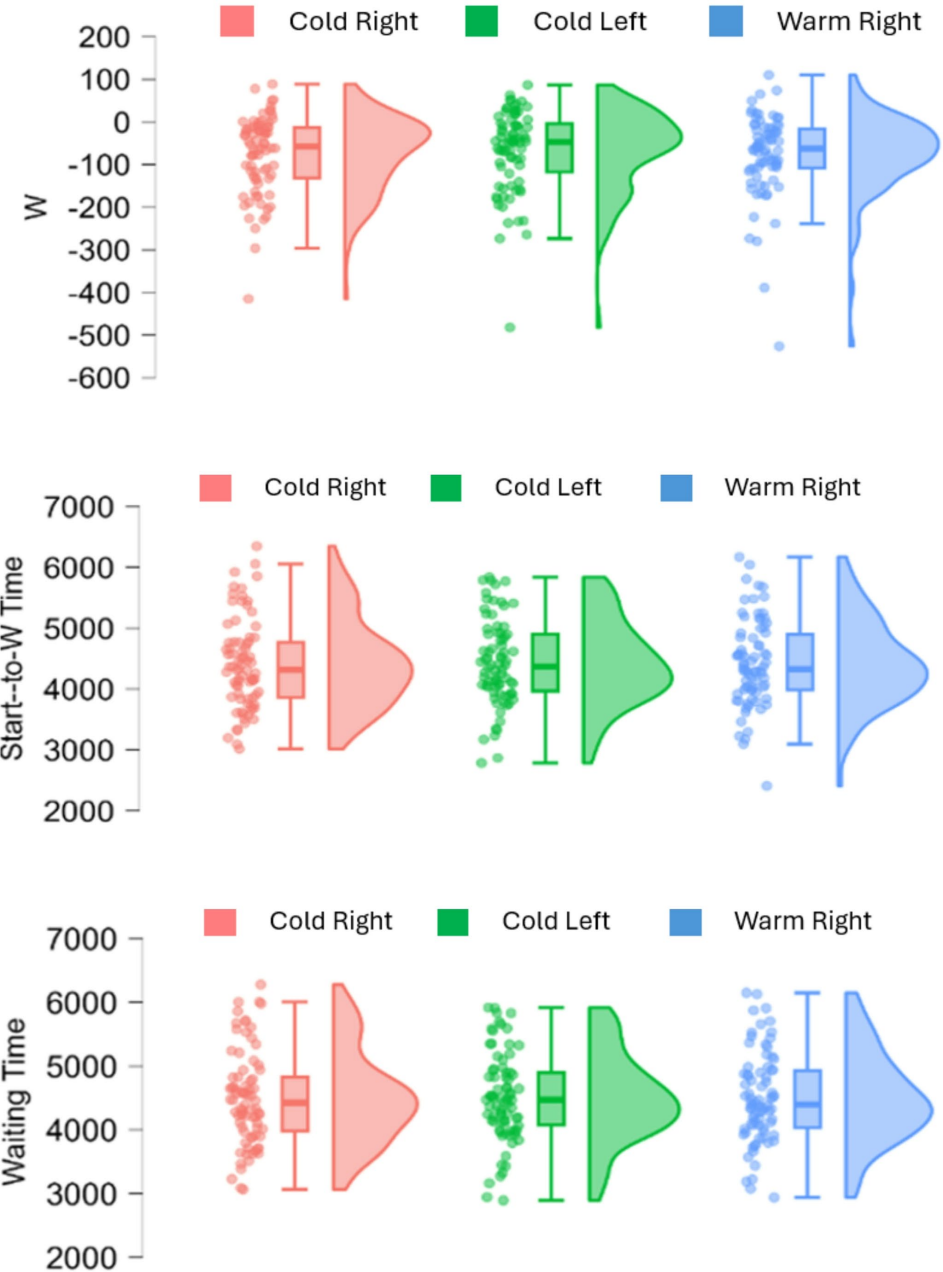
Raincloud Plots of the W Time (upper panel), Start-to-W Time (middle panel), and Waiting Time (lower panel). Cold Right Condition was highlighted in RED, Cold Left Condition was highlighted in GREEN, and Warm Right Condition was highlighted in BLUE. All results were presented in millisecond (ms). The figures were created via JASP (JASP Team, 2024; ver. 0.19.3)

Descriptive statistics are summarized in Table 1. In the pre-registered analyses, the Bayesian repeated measure ANOVA revealed strong support for the null hypothesis regarding the main effect of Condition (Cold Right, Cold Left, and Warm Right) across all three dependent measures (Start-to-W Time; BF10 = 0.05, W; BF10 = 0.06, and Waiting Time; BF10 = 0.03). Regarding the main effect of Order, Bayesian repeated measures ANOVA revealed moderate support to the null hypothesis in W (BF10 = 0.13), while no clear evidence was found in the Start-to-W Time (BF10 = 1.33) and Waiting Time (BF10 = 1.27). Importantly, Bayesian repeated measures ANOVA provided extreme support for the null hypothesis regarding the interaction effect between Condition and Order (the 6 counterbalance sequences) across all the three dependent measures (Start-to-W Time; BF10 = 0.002, W; BF10 = 0.006, and Waiting Time; BF10 = 0.004) (Fig. 5).

**Table 1:** Descriptive statistics

	Start-to-W Time		W		Waiting Time	
	M	S.E.	M	S.E.	M	S.E.
Cold Right	4413	84.4	-76.3	10.4	4489	83.9
Cold Left	4429	79.6	-67.4	11.0	4497	78.8
Warm Right	4423	81.4	-77.1	11.1	4500	79.7

The mean (M) and standard error (S.E.) are shown for each dependent measure. All results were presented in millisecond (ms)

### Exploratory analysis

#### Effect across time

In the exploratory analyses, the main effects of Time (First Half of Each Block, Second Half of Each Block) were extremely supported by the Bayesian repeated measure ANOVA for Start-to-W Time (BF10 = 1496) and Waiting Time (BF10 = 663), while the null hypothesis was moderately supported for W (BF10 = 0.15). Participants generated their intention and executed the action earlier in the first half of the block, regardless of the experimental condition. However, the Bayesian analysis revealed extreme support for the null hypothesis regarding the interaction between Condition and Time across all dependent measures (Start-to-W Time; BF10 = 0.006, W; BF10 = 0.003, and Waiting Time; BF10 = 0.005).

#### Effect across pain rating

We also conducted an analysis of pain ratings. The median pain rating was 5 (S.D.: 2.24, range: 0 to 9). Participants who rated their pain below 5 were categorized into the Low Pain group, while those who rated their pain above 5 were categorized into the High Pain group. The grouping was not perfectly balanced (Low Pain group: 37 participants, High Pain group: 31 participants).

Next, we conducted a Bayesian repeated measures ANOVA with the factors Condition (Cold Right, Cold Left, Warm Right) and Pain Rating (High Pain vs. Low Pain) to examine whether the effect of the cold-pressor task depended on pain intensity. Regarding the main effects, the null effect of Pain Rating was ambiguous across all dependent measures (Start-to-W Time: BF10 = 0.29, W: BF10 = 0.36, and Waiting Time: BF10 = 0.29). Importantly, the Bayesian analysis again provided extreme support for the null hypothesis regarding the interaction between Condition and Pain Rating across all dependent measures (Start-to-W Time: BF10 = 0.007, W: BF10 = 0.01, and Waiting Time: BF10 = 0.006).

#### Single-trial level model

The above analyses were conducted based on the mean values of the dependent measures for each participant. However, the potential effect of the cold-pressor task manipulation on our intentional action could also be revealed in other parameters of the dependent measures, such as the shape of individual distributions. We therefore fitted participants’ Waiting Time to the Shifted Wald distribution (Anders et al., 2016) and modelled the rate of signal accumulation (γ) and the decision threshold (α). For γ, the Bayesian repeated measure ANOVA revealed strong support for the null hypothesis regarding the main effect of Condition (BF10 = 0.05), and extreme support for the null hypothesis was found for the interaction effect between Condition and Time (BF10 = 0.007). Similarly, for α, the Bayesian repeated measure ANOVA revealed strong and very strong support for the null hypothesis regarding the main effect of Condition (BF10 = 0.03). Finally, extreme support to the null hypothesis was revealed for the interaction effect between Condition and Time (BF10 = 0.008). Taken together, the Bayesian evidence supported the idea that the cold-pressor task manipulation did not affect participants’ intentional action.

## Discussion

The current study aimed to investigate whether participants’ arbitrary decisions in a Libet-style experiment varied based on the somatosensory feedback in the afferent system. Participants performed the cold pressor task before conducting the Classical Libet task. The cold pressor task was used to induce a sense of numbness in the participants’ somatosensory system.

We hypothesized that somatosensory feedback in the afferent motor system is one of the parameters that guide participants’ arbitrary decisions. In this case, inducing numbness of the responding hand should disrupt the somatosensory feedback being accumulated, thus prolonging the time needed for the signal needs to cross the intention and action thresholds. This would predict an increase in the Start-to-W Time, W, and Waiting Time in the Cold Right Condition (in which the somatosensory feedback of the action hand was disrupted), compared to the Warm Right Condition.

However, the cold pressor task not only affected participants somatosensory feedback but also induced pain and stress to the recipient (Lovallo, 1975). Hence, any observed effects could potentially be due to the pain and/or stress experienced during the cold pressor task. To address this, participants performed the cold pressor task with both hands in separate experimental blocks (i.e., the Cold Right and Cold Left Conditions). Based on the tactile suppression effect that is specific to the effector hand, we hypothesized that the behavioral effect due to disruption of somatosensory feedback would be stronger, or even specific to, the cold pressor task applied to the acting hand (i.e., the Cold Right Condition), compared to the Cold Left Condition.

Contrary to our hypothesis, our Bayesian analyses provided conclusive evidence supporting the null hypothesis when comparing the three behavioral measures in the Classical Libet task (i.e., the Start-to-W Time, the W, and the Waiting Time) among the three experimental conditions (Cold Right Condition, Cold Left Condition, and the Warm Right Condition). One possible explanation for the null finding was that analyzing the individual averages of the behavioral measures across trials might not be sensitive enough to capture the behavioral difference among conditions. Previous studies (Schurger et al., 2012; Schurger, 2018; Trovò, 2021) successfully fitted the behavioural measures with signal accumulation models. Under the signal accumulation model, it was possible that participants’ decision signals were weakened in an experimental condition, but they also lowered their decision threshold, resulting in similar averages of the behavioral measures across conditions. To address this possibility, we explored whether participants’ accumulation rates and decision thresholds varied across experimental conditions. Consistent with our pre-registered analyses reported above, the Bayesian analyses demonstrated conclusive evidence supporting the null hypothesis in comparing the signal accumulation rate and decision threshold across the three experimental conditions.

Since the current findings supporting the null hypothesis were apparently contrary to our predictions, some possible explanations are warranted. The strongest interpretation is that while other body signals, such as respiration (Park et al., 2020, 2022) and cardiac phases (Kunzendorf et al., 2019), contribute to arbitrary intentional action, somatosensory feedback does not contribute to intentional action. However, we consider this claim to be too strong to make based on the current study, as it is impossible to assert the absence of an effect based on one single experiment, even though the Bayesian analysis strongly supported the null hypothesis across all behavioral measures. Instead, we propose that the role of somatosensory feedback in arbitrary decisions in Libet-style experiment is not as prominent as we initially hypothesized, such that a mild manipulation to it could not affect the behavioral markers of arbitrary decisions (i.e., the Start-to-W time, the W, and the Waiting Time). This, of course, does not entirely dismiss the role of somatosensory feedback in arbitrary decisions. We, on the other hand, suggest that different body signals may have different weights in their contributions to the generation of intentional action, and the weight of somatosensory feedback is relatively low. To verify this, further studies may integrate manipulations to different interoceptive signals, including respiratory signal, cardiac signal, and somatosensory feedback signal, to model the weight of their contributions to the behavioral markers of arbitrary decisions. This will provide a holistic picture on how interoceptive signals can contribute to arbitrary decisions and help specify the decision parameters involved. We invite future research to further investigate this.

Another related yet different possibility is that the disruption of somatosensory feedback redistributes the load of different signals being accumulated, rather than simply delaying arbitrary decisions. In another line of research, previous studies (Atkins et al., 2001; Case et al., 2013) suggested that humans can flexibly compare the relative reliability of different signals and adjust the weight of these signals accordingly to better accomplish tasks. Similarly, under the integration-to-bound model, participants integrate different interoceptive signals to guide their decisions in arbitrary contexts, such as in the Libet-style experiment. If participants somatosensory feedback was disrupted, they might simply place less weight on the somatosensory feedback and redistribute that weight to other interoceptive signals. Based on this explanation, one should expect the coupling between intentional action and respiratory/cardiac cycle (Kunzendorf et al., 2019; Park et al., 2020, 2022) should be enhanced when somatosensory feedback is disrupted. While we cannot test this hypothesis, as we did not measure respiratory/cardiac signal in the current experiment, we encourage future research to investigate this.

An alternative explanation for the unexpected finding is that the manipulation we used may not induce enough change in the somatosensory feedback to affect arbitrary intentional action. We considered the water temperature to be sufficiently cold based on our initial hypothesis, as a previous study by Dizmen and colleagues (2015) suggested that a cold-water bath in 10 °C could already lower participant’s performance in the O’Connor dexterity test and reaction time tests responding to visual and auditory stimuli. However, as mentioned in the last paragraph, the contribution of somatosensory feedback signal to intentional action was weaker than we have expected. Hence, it is possible that the expected effect can only be observed with an even lower water temperature. This limitation of the current study can be addressed in future research.

Another possibility is that the three behavioral measures that we used (i.e., the Start-to-W time, the W, and the Waiting Time) were not sensitive enough to capture the expected effect across conditions in our paradigm. While these three measures have successfully captured behavioral effects in other studies (e.g., Dominik et al., 2017; Ivanof et al., 2022; Shum et al., 2024), neural measures such as fMRI (e.g., Brass & Haggard, 2007; Kühn et al., 2009) and EEG (e.g., Park et al., 2020;2022; Rigoni et al., 2011; Trovò et al., 2021; Walsh et al., 2010) have also been used in other studies to provide different perspectives and additional information regarding how the biological system reacts to experimental manipulations in arbitrary intentional action. Future research may explore whether the effect of somatosensory feedback on arbitrary intentional action can be observed in the neural measures.

Besides the main research question, an additional interesting observation is that behavioral parameters in intentional action were apparently not affected by the subjective pain. We drew this conclusion by two analyses. First, the null effects were supported in the Bayesian repeated measures ANOVA among the three conditions for all behavioral parameters, suggesting that participants did not behave differently in the only condition where they did not experience any pain (i.e., the Warm Right condition). Second, participants’ pain rating did not modulate the effect of the cold-pressor manipulation on behavioral parameters. Contrary to our current findings, previous studies (Misra et al., 2017; Perini & Bergstrand, 2013) suggested that participants overall reacted faster when they were experiencing ongoing pain. Misra et al. (2017) proposed that this reduction in reaction time could potentially be explained by pain-related suppression of beta-power in the contralateral premotor and supplementary motor areas, which are also crucial for performing arbitrary decisions (Brass & Haggard, 2008).

Why is there such a contradiction? One possibility is that the pain elicited by our manipulation was not strong enough to suppress the beta-power, so the intentional action was not affected. Although we did not measure participants’ EEG signal, we considered this explanation to be unlikely. Some participants reported high or even extreme scores in the pain rating (6 to 9 out of 10). The manipulation should be at least strong enough to them and they should demonstrate a faster Waiting Time. However, our Bayesian hypothesis neither supported the main effect of pain rating nor the interaction between the pain rating and the experimental condition, indicating that these participants were not different from those who experienced less pain. Another possibility lies in the difference in experimental paradigm. Specifically, the paradigms that Misra et al. (2017) and Perini and Bergstrand (2013) used were cued response paradigm, which are inherently different from the Libet-style experiment where participants are required to decide arbitrarily.

The last possibility deserves special attention: the potential effect of numbness (which we hypothesize to slow down intentional action) and the effect of pain (which presumably speeds up intentional action) may have cancelled each other out, resulting in unchanged waiting times among conditions. This explanation not only accounts for the null effect between the Cold Right and the Warm Right Condition, but also potentially explains the null effect between the Cold Right and the Cold Left conditions. As suggested by Misra et al. (2017), pain-related suppression of beta-power in the premotor and supplementary motor area was bilateral. This means that in both the Cold Left Condition and Cold Right conditions, participant’s beta-power in the left premotor area (which is contralateral to the moving hand) should be lowered, leading to a faster execution of movement. In our initial hypothesis, we assumed the effect of numbness to intentional action to be specific to the hand put under the cold water, predicting a difference between the Cold Left and Cold Right conditions. However, if the effect of numbness is also bilateral, then the Cold Left and Cold Right conditions would no longer be different from each other, resulting in the null effects observed.

Aside from the mutual cancellation of effects, another limitation of our study is that the effects of pain and numbness were intertwined in the manipulation. Although our control condition (i.e., the Cold Left condition) successfully accounted for the general effect from the cold pressor task on our body (e.g., the increase of heart rate and stress level), it did not control for the side effects specific to the effector (e.g., the pain to the effector). In this case, even if we could observe an effect in the comparison between the Cold Right condition and the Cold Left condition, one might argue that we could not disentangle the the influence of pain from that of numbness in the effector. While we acknowledge this possibility, it is also crucial to keep in mind that a number of these unspecific effects such as pain dissipate relatively soon after participants remove the hand from the cold water. Nevertheless, future studies investigating similar topics are still encouraged to design manipulations that more clearly dissociate pain and numbness components.

In summary, the current study investigated whether manipulating participants’ somatosensory feedback in their motor systems affected their arbitrary decision in Libet-style experiment. Our Bayesian analysis results overall supported the null hypothesis, and we have provided some potential explanations for this null finding. Among them, the most likely explanation is that somatosensory feedback from our afferent motor system does not play a major role in arbitrary decision in Libet-style experiment compared to other interoceptive signals, so a moderate manipulation of our somatosensory feedback does not affect those decisions. Despite this unexpected finding, this research is the first attempt to investigate dynamic, bi-directional relationship between the brain and the motor system in the Libet-style experiment. This study lays the foundation for this research avenue, and we have outlined future directions to establish this area as a substantial component of our understanding about intention and intentional action.

## Supplementary Information

Below is the link to the electronic supplementary material.

Supplementary Material 1 (DOCX 33.8 KB)

## Data Availability

All data, analysis scripts, and experiment programs are available on the Open Science Framework (OSF; https://osf.io/vduar/?view_only=fd0614f10e1e4c3b9d6d111de6ac6021). All participants agreed us to share the data in anonymous form.
