# Supplementary material for: Moderate manipulation to somatosensory feedback does not affect Libet-style intentional action
Source: Psychol Res. 2025 Sep 8;89(5):140. doi: 10.1007/s00426-025-02178-1 (PMC12420747; doi:10.1007/s00426-025-02178-1)
Supplement: Supplementary file 1 — Supplementary Material 1 (DOCX 33.8 KB) [file 426_2025_2178_MOESM1_ESM.docx]

**Supplementary Materials**

In this section, we are reporting our pre-registered analyses in the Frequentist approach. In the repeated measure ANOVA, the main effect of Condition (Cold Right, Cold Left, and Warm Right) was not statistically significant in the Start-to-W Time (F(2, 144) = 0.12, p = .888, partial eta square, ŋ^2^p = 0.002) , the W (F(2, 144) = 0.72, p = .487, ŋ^2^p = 0.010), and the Waiting Time (F(2, 144) = 0.03, p = .975, ŋ^2^p = 0.00). The main effect of Order (the 6 counterbalance sequences) was not statistically significant for W (F(2, 144) = 0.60, p = .699, ŋ^2^p = 0.040). However, it was statistically significant in the Start-to-W Time (F(2, 144) = 2.54, p = .036, partial eta square, ŋ^2^p = 0.150) and the Waiting Time (F(2, 144) = 2.53, p = .037, ŋ^2^p = 0.149). Post-hoc comparison with Bonferroni correction revealed that participants who performed the Warm Right Condition first, followed by the Cold Left Condition and then the Cold Right Condition, intended and acted significantly slower than those who conducted the Cold Left Condition first, followed by the Cold Right Condition, and then the Warm Right Condition at last (Start-to-W Time: t(72) = 3.45, p_Bonferroni_ = .012; Waiting Time: t(72) = 3.51, p_Bonferroni_ = .012). Importantly, the interaction effect between Condition and Order was also not statistically significant in all the three behavioral measures (Start-to-W Time: F(10, 144) = 0.39, p = .951, ŋ^2^p = 0.026; W: F(10, 144) = 1.18, p = .309, ŋ^2^p = 0.076; Waiting Time: F(10, 144) = 0.60, p = .812, ŋ^2^p = 0.040).

In the repeated measure ANOVA with effects of Condition (Cold Right, Cold Left, and Warm Right) and Time (First Half of Each Block, Second Half of Each Block), the main effect of Time (First Half of Each Block, Second Half of Each Block) was statistically significant in Start-to-W Time (F(1, 77) = 67.2, p < .001, ŋ^2^p = 0.466) and Waiting Time (F(1, 77) = 61.2, p < .001, ŋ^2^p = 0.443). Similarly, the main effect of Time was also statistically significant in W (F(1, 77) = 8.48, p = .005, ŋ^2^p = 0.099). However, the interaction effect between Condition and Time was not significant in any of the dependent measures (Start-to-W Time: F(2, 154) = 1.05, p = .351, ŋ^2^p = 0.014; W: F(2, 154) = 0.20, p = .817, ŋ^2^p = 0.003; Waiting Time: F(2, 154) = 0.93, p = .397, ŋ^2^p = 0.012). Post-hoc test (Start-to-W Time: t(77) = -8.20 ; W: t(77) = -2.91; Waiting Time: t(77) = -7.82; all in the contrast First Half – Second Half) revealed that participants generated their intention and executed the action earlier in the first half of the block, regardless of the experimental condition.

We also repeated our exploratory analysis in Frequentist approach. Regarding the analysis with the pain rating, repeated measures ANOVA with the factors Condition (Cold Right, Cold Left, Warm Right) and Pain Rating (High Pain vs Low Pain) revealed insignificant results in the main effect of Pain Rating across all dependent measures (Start-to-W Time: F(1, 66) = 0.004, p = .949, ŋ^2^p < 0.001; W: F(1, 66) = 1.28, p = .261, ŋ^2^p = 0.019; Waiting Time: F(1, 66) = 0.93, p = .397, ŋ^2^p < 0.001). Aligned with the Bayesian analysis, the interaction term between Condition and Pain Rating across all dependent measures was also not statistically significant (Start-to-W Time: F(2, 132) = 0.15, p = .861, ŋ^2^p = 0.002; W: F(2, 132) = 0.27, p = .765, ŋ^2^p = 0.004; Waiting Time: F(2, 132) = 0.09, p = .910, ŋ^2^p = 0.001).

Finally, we conducted repeated measure ANOVA with the model parameters that we obtained based on the Shifted Wald distribution (Anders et al., 2016; see Methods for details). We modelled the rate of signal accumulation (γ) and the decision threshold (α) based on Waiting Time. For γ, the main effect of Condition in the repeated measure ANOVA was not statistically significant (F(2, 154) = 0.79, p = .455, ŋ^2^p = 0.010). The main effect of Time was significant (F(1, 77) = 5.24, p = .025, ŋ^2^p = 0.064). More importantly, the interaction effect between Condition and Time was not significant (F(2, 154) = 0.14, p = .867, ŋ^2^p = 0.002). Post-hoc test (t(77) = -2.29 in the contrast First Half – Second Half) indicated that the signal accumulation rate was higher in the second half of the block, regardless of the experimental condition.

For α, the main effect of Condition in the repeated measure ANOVA was not statistically significant (F(2, 154) = 0.48, p = .622, ŋ^2^p = 0.006). The main effect of Time was significant (F(1, 77) = 12.1, p < .001, ŋ^2^p = 0.136). More importantly, the interaction effect between Condition and Time was not significant (F(2, 154) = 0.29, p = .749, ŋ^2^p = 0.004). Post-hoc test (t(77) = -3.48 in the contrast First Half – Second Half) indicated that the threshold rate was higher in the second half of the block, regardless of the experimental condition. Overall, the results were consistent with those in the Bayesian approach, such that there was no evidence supporting the cold-pressor manipulation for affecting any behavioral parameters in the Libet task.
